# Synergistic effects of isomorellin and forbesione with doxorubicin on apoptosis induction in human cholangiocarcinoma cell lines

**DOI:** 10.1186/1475-2867-14-68

**Published:** 2014-10-24

**Authors:** Chariya Hahnvajanawong, Wareeporn Wattanawongdon, Chariya Chomvarin, Natthinee Anantachoke, Sakawrat Kanthawong, Banchob Sripa, Vichai Reutrakul

**Affiliations:** Department of Microbiology, Center of Excellence for Innovation in Chemistry, Faculty of Medicine, Khon Kaen University, Khon Kaen, 40002 Thailand; Liver Fluke and Cholangiocarcinoma Research Center, Khon Kaen University, Khon Kaen, 40002 Thailand; Department of Pharmacognosy, Faculty of Pharmacy, Mahidol University, Bangkok, 10400 Thailand; Department of Pathology, Faculty of Medicine, Khon Kaen University, Khon Kaen, 40002 Thailand; Department of Chemistry, Faculty of Science, Mahidol University, Bangkok, 10400 Thailand

**Keywords:** Isomorellin, Forbesione, Doxorubicin, Synergistic effect, Apoptosis, Human cholangiocarcinoma cell lines

## Abstract

**Background:**

Chemotherapy for advanced cholangiocarcinoma (CCA) is largely ineffective, but innovative combinations of chemotherapeutic agents and natural compounds represent a promising strategy. In our previous studies, isomorellin and forbesione, caged xanthones isolated from *Garcinia hanburyi*, were found to induce cell cycle arrest and apoptosis in CCA cell lines. The subject of our inquiry is the synergistic effect(s) of these caged xanthones with doxorubicin on growth inhibition and apoptosis induction in human CCA cell lines.

**Methods:**

KKU-100, KKU-M139 and KKU-M156 cell lines and Chang cells were treated with either isomorellin or forbesione alone or in combination with doxorubicin. Cell viability was determined using the sulforhodamine B assay. The combined effects of plant compounds with doxorubicin were analyzed using the isobologram and combination index method of Chou-Talalay. Apoptosis was determined by ethidium bromide/acridine orange staining. Protein expressions were determined by Western blot analysis.

**Results:**

Isomorellin or forbesione alone inhibited the growth of these CCA cell lines in a dose-dependent manner and showed selective cytotoxicity against CCA cells but not against Chang cells. Isomorellin/doxorubicin combination showed a synergistic growth inhibitory effect on KKU-M139 and KKU-M156 cells, while the forbesione/doxorubicin combination showed a synergistic growth inhibitory effect on KKU-100 and KKU-M139 cells. The percentages of apoptotic cells were significantly higher in the combined treatments than in the respective single drug treatments. The combined treatments strongly enhanced the expression of Bax/Bcl-2, activated caspase-9 and caspase-3, while suppressing the expression of survivin, procaspase-9 and procaspase-3, compared with single drug treatments. The degree of suppression of NF-κB activation mediated by a decrease in the expression of NF-κB/p65, a reduction of the pIκB-α level and an increase in the IκB-α protein level, was significantly higher in the combined treatment groups than in the single drug treatment groups. The degree of suppression of MRP1 protein expression was also significantly higher in the combined treatment than in the single drug treatment groups.

**Conclusion:**

The combinations of isomorellin/doxorubicin and forbesione/doxorubicin showed significant synergistic effects on the growth inhibition and apoptosis induction in KKU-M156 and KKU-100 cells. Caged xanthones may be useful adjunct treatments with chemotherapy for *Opisthorchis viverrini* (OV)-associated CCA.

## Background

Cholangiocarcinoma (CCA) is a tumor arising from the bile duct epithelium. The worldwide incidence and mortality rate of CCA are increasing [1, 2]. This cancer has a poor prognosis because the majority of patients are diagnosed at a late stage and the tumor often recurs after surgery [3]. The treatment option for advanced CCA is chemotherapy [4]. Doxorubicin is one of the chemotherapeutic drugs used to treat CCA [4], but it is known to (a) be highly toxic to normal tissues; (b) cause immunosuppression and secondary cardiomyopathy; and (c) engender the drug resistance of tumor cells, thereby limiting its efficacy [5, 6]. Chemotherapeutic drugs induce apoptosis through DNA damage and cell cycle arrest. However, defects in oncogene activation and/or the deregulation of apoptotic signaling pathways are common in cancer cells, allowing them to evade apoptosis [7]. In CCA, increased expression levels of Bcl-2 and survivin (anti-apoptotic proteins) are frequently observed and are associated with tumor progression and aggressiveness [8, 9], while the overexpression of Mcl-1 and survivin is associated with drug resistance [10, 11]. The activation of nuclear factor-kappa B (NF-κB), a key transcriptional regulator of genes involved in cell survival, proliferation and apoptosis induction, was found to be associated with CCA development in an animal model [12], and high expression levels of NF-κB in CCA patient tissues have also been reported [Seubwai et al., manuscript in preparation]. Enhanced expression of the ATP-binding cassette transporters of the multidrug resistance protein families MDR and MDR-associated proteins (MRPs) in cancer cells is a major cause of multidrug resistance both *in vitro* and *in vivo*[13]. The P-glycoprotein (P-gp) and MRP1-3 are expressed in cholangiocytes [14, 15], and their overexpression is associated with drug resistance in CCA. Consequently, alternative adjunct therapies for CCA are urgently needed.

Plant-derived caged xanthones are potential adjunct therapies to existing chemotherapeutic strategies. Gambogic acid, a caged xanthone, was reported to overcome the docetaxel resistance of gastric cancer BGC-823/Doc cells [16] and to enhance 5-fluorouracil (5-FU)-induced apoptosis in human gastric cancer BGC-823 cells [17]. Han and Xu [18] reported that, among the caged xanthones isolated from *Garcinia hanburyi*, gambogic acid is the most extensively studied compound, with promising anticancer effects against a variety of cancer cell lines both *in vitro* and *in vivo*[19, 20]. Multiple mechanisms have been proposed for gambogic acid-induced anticancer activities, including the induction of apoptosis [19], suppression of telomerase activity [21], induction of cell-cycle arrest [22], inhibition of cancer metastasis and angiogenesis [20, 23], inhibition of topoisomerase IIα activity [24] and inhibition of the NF-κB signaling pathway [25]. In various animal models, gambogic acid is well tolerated, suggesting the existence of a therapeutic approach for its use [26, 27]. Gambogic acid injection was recently approved by the Chinese State Food and Drug Administration as an antitumor treatment and will enter phase II clinical trials [28].

We report that four caged xanthones; isomorellin, gambogic acid, forbesione and isomorellinol, have a selective inhibitory effect against the growth of the CCA cell lines KKU-100 and KKU-M156. These caged xanthones induce mitochondrial apoptosis pathways [29] and induce cell cycle arrest at G0/G1 phase [30]. These results suggest that caged xanthones could be used in combination therapy due to their low toxicity to normal cells and multiple targeted effects [29, 30].

To date, the effects of caged xanthones combined with conventional chemotherapeutic drugs have not been evaluated for the treatment of human CCA. Therefore, we examined the effects of combining caged xanthones (isomorellin and forbesione) with doxorubicin compared with their individual activities against CCA KKU-100, KKU-M139 and KKU-M156 cells *in vitro*. We also investigated the possible mechanisms of any observed synergistic effects.

## Results

### Inhibition of cholangiocarcinoma cell growth by isomorellin, forbesione and doxorubicin

**Figure 1:**
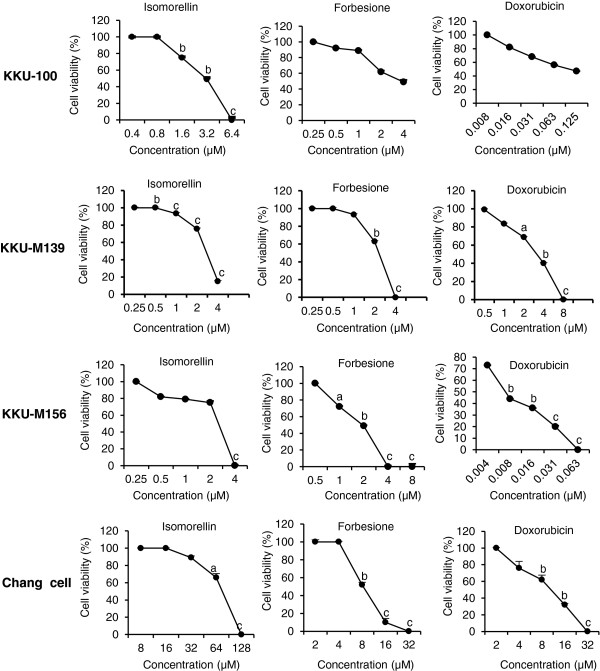
**Growth inhibitory effects of caged xanthones and doxorubicin on KKU-100, KKU-M139, KKU-M156 and Chang cells.** Cells were treated with DMSO or with the indicated concentrations of isomorellin, forbesione and doxorubicin for 72 h. Data are presented as the mean ± SE of three independent experiments. ^a^ *P* < 0.05, ^b^ *P* < 0.01, ^c^ *P* < 0.001 *vs.* DMSO-treated cells.

The inhibitory effects of isomorellin, forbesione and doxorubicin on the growth of CCA cell lines and Chang cells were evaluated using the sulforhodamine B (SRB) assay. After exposure to these compounds and doxorubicin, the growth of KKU-100, KKU-M139, KKU-M156 and Chang cells was inhibited in a dose-dependent manner (Figure 1). The IC_50_ value of isomorellin in Chang cells was 22.2-, 27.4- and 32.8-fold higher than that in KKU-100, KKU-M139 and KKU-M156 cells, respectively (Table 1). Similarly, the IC_50_ value of forbesione in Chang cells was 3.7-, 5.7- and 4.9-fold higher than that in KKU-100, KKU-M139 and KKU-M156 cells, respectively (Table 1). These results indicate a selective growth suppression effect of these compounds on cancer cells.

**Table 1:** **IC**_**50**_ **values of the caged xanthones and doxorubicin in cholangiocarcinoma cells and Chang cells**

Compounds	IC _50_ value (μM)			
	KKU-100	KKU-M139	KKU-M156	Chang
Isomorellin	3.34 ± 0.12	2.71 ± 0.10	2.26 ± 0.05	74.19 ± 0.39
Forbesione	3.53 ± 0.05	2.29 ± 0.04	2.63 ± 0.05	13.13 ± 0.57
Doxorubicin	0.06 ± 0.02	3.74 ± 0.08	0.007 ± 0.002	13.65 ± 0.41

Mean ± SE of three independent experiments.

When KKU-100, KKU-M139 and KKU-M156 cells were treated with isomorellin, forbesione or doxorubicin, the IC_50_ values for each compound were different, and KKU-M156 cells were more susceptible than KKU-100 and KKU-M139 cells to all three compounds (Table 1). The dose ranges of isomorellin and/or doxorubicin or forbesione and/or doxorubicin were determined for further study in accordance with the IC_50_ values for each cell line.

### Effects of isomorellin or forbesione in combination with doxorubicin on cell growth

**Figure 2:**
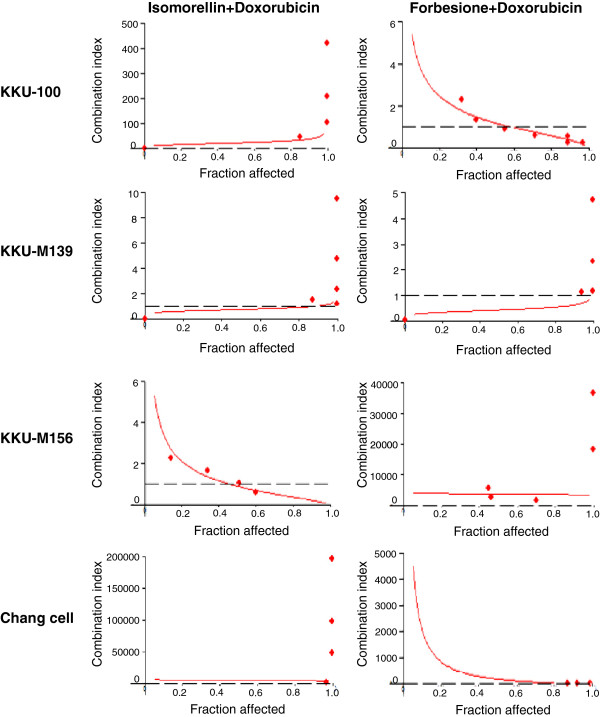
**Caged xanthones and doxorubicin combination toxicity.** KKU-100, KKU-M139, KKU-M156 and Chang cells were treated with the appropriate concentrations of isomorellin and doxorubicin or forbesione and doxorubicin for 72 h. Combination index (CI) *vs.* fraction affected (*fa*) plots were obtained from the median-effect analysis program (CalcuSyn, Biosoft, Cambridge, UK). Dashed lines indicate a CI of 1. Each value represents the mean of three independent experiments.

The combined effects of isomorellin/doxorubicin or forbesione/doxorubicin on the growth inhibition of KKU-100, KKU-M139, KKU-M156 and Chang cells were determined using isobologram analysis [31, 32]. The proxies for the combined effects were (a) the dose reduction index (DRI), (b) the combination index (CI) and (c) the dose-effect levels of cell growth inhibition at the 50%, 75% and 90% inhibitory concentrations (IC_50_, IC_75_ and IC_90_) (Table 2). The combination of isomorellin/doxorubicin in KKU-M156 and KKU-M139 cells was synergistic, but this combination was antagonistic in KKU-100 cells. The combination of isomorellin/doxorubicin in KKU-M156 cells was synergistic at all three dose levels, with respective CIs of 0.92, 0.47 and 0.24 (Figure 2 and Table 2). The combination of isomorellin/doxorubicin showed a synergistic inhibitory effect on the growth of KKU-M139 cells at low and medium concentrations (IC_50_ and IC_75_), with respective CIs of 0.80 and 0.92 (Figure 2 and Table 2). The combination of forbesione/doxorubicin in KKU-100 and KKU-M139 cells was synergistic, but the combination was antagonistic in KKU-M156 cells. The combination of forbesione/doxorubicin in KKU-100 cells was synergistic at medium and high concentrations (IC_75_ and IC_90_) with CIs of 0.69 and 0.39, respectively (Figure 2 and Table 2). The combination of forbesione/doxorubicin showed a synergistic inhibitory effect on the growth of KKU-M139 cells at all three doses, with CIs of 0.46, 0.55 and 0.65, respectively (Figure 2 and Table 2). Both combinations of isomorellin/doxorubicin or forbesione/doxorubicin were antagonistic in Chang cells at all doses examined (CI > 1) (Figure 2).

**Table 2:** **Dose-effect relationships of isomorellin, forbesione and doxorubicin combinations in cholangiocarcinoma cell lines**

Cell line	Single compounds, drug & combinations	Parameter	CI value			DRI value		
		***r***	IC _50_	IC _75_	IC _90_	IC _50_	IC _75_	IC _90_
KKU-M156	Isomorellin	0.86				29.65	53.54	96.69
	Doxorubicin	0.91				1.12	2.18	4.23
	(D)1 + (D)2 (1 : 1.25)	0.97	0.92	0.47	0.24			
KKU-100	Forbesione	0.88				4.46	8.32	15.51
	Doxorubicin	0.88				1.00	1.73	2.99
	(D)1 + (D)2 (1 : 0.25)	0.98	1.21	0.69	0.39			
KKU-M139	Isomorellin	0.93				2.15	1.89	1.66
	Doxorubicin	0.94				3.04	2.57	2.17
	(D)1 + (D)2 (2 : 1)	0.81	0.80	0.92	1.06			
	Forbesione	0.89				3.28	2.83	2.44
	Doxorubicin	0.89				6.57	5.20	4.12
	(D)1 + (D)2 (2 : 1)	0.85	0.46	0.55	0.65			

Dose-effect relationships calculated using the median-effect equation. The *r* linear correlation is the coefficient of the median effect plot (indicates conformity of data). CIs were calculated using Chou and Talalay’s CI equation. The DRI dose reduction index was measured by comparing doses required to reach a given degree of inhibition when using compound(s) or drug as a single agent and/or in combination.

As a result of the observed synergy between caged xanthones and doxorubicin, there was a considerable reduction in the DRI (Table 2). At dose levels corresponding to synergistic drug combinations, the DRI indicated that the doxorubicin concentration needed to inhibit 90% of the growth of cancer cells (IC_90_) could be decreased 4.23-fold (KKU-M156, isomorellin/doxorubicin), 2.99-fold (KKU-100, forbesione/doxorubicin), 2.17-fold (KKU-M139, isomorellin/doxorubicin) and 4.12-fold (KKU-M139, forbesione/doxorubicin) (Table 2).

### Synergistic effects of isomorellin/doxorubicin and forbesione/doxorubicin combinations on the induction of apoptosis in CCA cell lines

**Figure 3:**
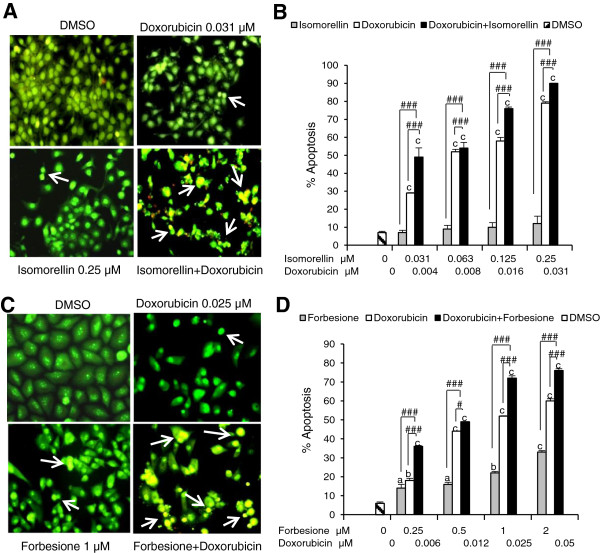
**Apoptosis induction by two caged xanthones and/or doxorubicin in cholangiocarcinoma (CCA) cell lines. A**, Morphological changes of KKU-M156 cells after treatment with DMSO, isomorellin (0.25 μM) and/or doxorubicin (0.031 μM) for 48 h. The cells were stained with ethidium bromide and acridine orange (EB/AO) (400X). The apoptotic cells with condensed chromatin or fragmented chromatin are indicated with arrows. **B**, Percentage of apoptotic cells in KKU-M156 cells treated with isomorellin and/or doxorubicin at the indicated concentration for 48 h. **C**, Morphological changes of KKU-100 cells after treatment with DMSO, forbesione (1 μM) and/or doxorubicin (0.025 μM) for 48 h. The cells were stained with EB/AO. **D**, Percentage of apoptotic cells in KKU-100 cells treated with forbesione and/or doxorubicin at the indicated concentration for 48 h. Data expressed as the mean ± SE (*n* = 3). ^a^ *P* < 0.05, ^b^ *P* < 0.01, ^c^ *P* < 0.001 *vs.* DMSO-treated cells. Comparison between single drug treatment and combined drug treatment is indicated by ^#^ *P* < 0.05, ^#*##*^ *P* < 0.001.

In our previous study [29], isomorellin and forbesione were found to induce apoptosis in KKU-100 and KKU-M156 cell lines through the mitochondrial pathway. When the combined effects of isomorellin/doxorubicin or forbesione/doxorubicin on the inhibition of growth in KKU-100, KKU-M139, KKU-M156 and Chang cells were examined, the highest synergistic effect was observed in KKU-M156 cells treated with the isomorellin/doxorubicin combination (CI value at IC_90_ = 0.24) and in KKU-100 cells treated with the forbesione/doxorubicin combination (CI value at IC_90_ = 0.39) (Table 2). To examine the synergistic effects of the isomorellin/doxorubicin and forbesione/doxorubicin combinations on the induction of apoptosis in CCA cell lines, KKU-M156 cells were treated with isomorellin and/or doxorubicin, while KKU-100 cells were treated with forbesione and/or doxorubicin at concentrations that demonstrated a synergistic effect. The apoptotic cells were determined by EB/AO staining and the percentage of apoptotic cells of 500 cells was calculated. Treatment with isomorellin, forbesione or doxorubicin alone increased apoptosis in both CCA cell lines, and the combination treatment augmented apoptosis in both groups (Figures 3A and C). The percentage of apoptotic cells was significantly higher in the isomorellin/doxorubicin-treated KKU-M156 cells compared to cells treated with either drug alone (Figure 3B). Similarly, a significantly higher percentage of apoptotic cells were observed in the forbesione/doxorubicin-treated KKU-100 cells than in cells treated with either drug alone (Figure 3D).

### Isomorellin/doxorubicin and forbesione/doxorubicin treatments modulate the expression of apoptosis regulating protein in CCA cell lines

**Figure 4:**
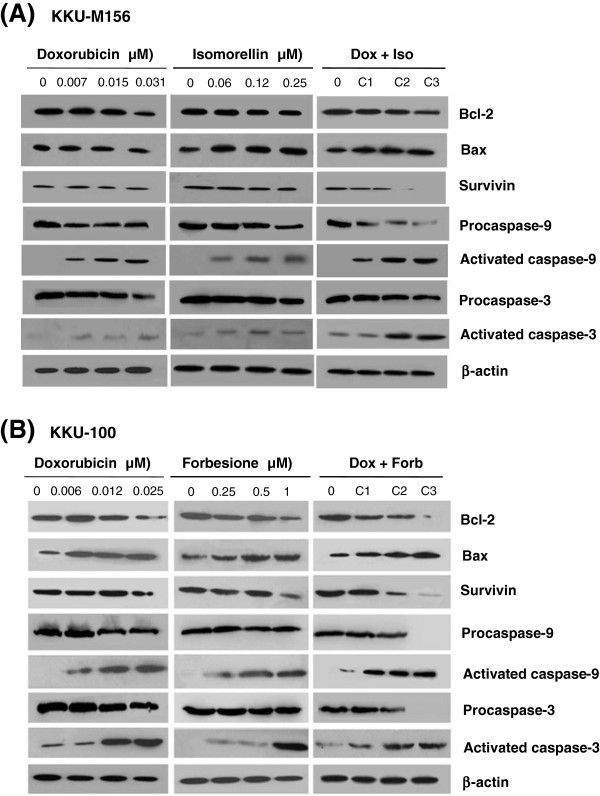
**Effect of isomorellin, forbesione and/or doxorubicin on apoptosis regulated protein expression in CCA cells. A**, KKU-M156 cells treated with DMSO or indicated concentrations of doxorubicin or isomorellin or the combination of doxorubicin + isomorellin (C1: 0.007 + 0.06 μM, C2: 0.015 + 0.12 μM, C3: 0.031 + 0.25 μM). **B**, KKU-100 cells treated with DMSO or indicated concentrations of doxorubicin or forbesione or the combination of doxorubicin + forbesione (C1: 0.006 + 0.25 μM, C2: 0.012 + 0.5 μM, C3: 0.025 + 1 μM) for 48 h. Protein expression was determined by Western blot analysis.

To determine the possible molecular mechanisms of the synergistic induction of apoptosis in cells treated with the isomorellin/doxorubicin and forbesione/doxorubicin combinations, the expression levels of mitochondrial apoptosis regulating proteins were determined using Western blot analysis. When KKU-M156 cells were treated with either isomorellin or doxorubicin or a combination of these two drugs, the expression of Bcl-2 protein was significantly decreased, whereas Bax protein expression was significantly increased in a dose-dependent manner, particularly in cells treated with the combination of both drugs (Figure 4A). Bcl-2 expression decreased by 0.41-fold in cells treated with combination 3 (C3) (0.031 μM doxorubicin + 0.25 μM isomorellin), which was significantly less than that of cells treated with 0.031 μM doxorubicin (0.82-fold) or with 0.25 μM isomorellin (0.76-fold) (Table 3). In contrast, the increase in Bax expression in cells treated with C3 was 2.13-fold compared to the control cells, and this increase was significantly higher than that of cells treated with doxorubicin (1.45-fold) or isomorellin (1.32-fold) alone. Likewise, the ratio of Bax/Bcl-2 increased significantly, especially in the combination treatment group (Table 3).

**Table 3:** **Fold-change of proteins in isomorellin- and/or doxorubicin-treated cells** ***vs.*** **DMSO-treated KKU-M156 cells**

		KKU-M156 cell		
Proteins	Treatments	Fold-changes of proteins in treated ***vs.*** control cells		
		Concentration of isomorellin or doxorubicin (μM)		
	Isomorellin	0.06	0.12	0.25
	Doxorubicin	0.007	0.015	0.031
	Iso + Dox	C1	C2	C3
Bcl-2	Isomorellin	0.83^a^	0.83^a^	0.76^a^
	Doxorubicin	1.00	0.95	0.82^a^
	Iso + Dox	0.84^a^	0.80^b,–,*^	0.41^c,*,*^
Bax	Isomorellin	1.26	1.32^c^	1.32^c^
	Doxorubicin	1.20	1.33^c^	1.45^c^
	Iso + Dox	1.84^b^	1.93^c,*,*^	2.13^c,*,*^
Bax/Bcl-2	Isomorellin	0.48^c^	0.50^b^	0.50^b^
	Doxorubicin	0.35^c^	0.40^b^	0.51^b^
	Iso + Dox	0.62^c^	1.09^–,*,*^	2.35^c,*,*^
Survivin	Isomorellin	1.00	0.96	0.86
	Doxorubicin	0.96	0.96	0.95
	Iso + Dox	0.96	0.64^b,*,*^	0.02^c,*,*^
Procaspase-9	Isomorellin	0.88	0.84	0.74^a^
	Doxorubicin	0.87	0.82	0.76^a^
	Iso + Dox	0.82^a^	0.57^b,*,*^	0.39^b,*,*^
Activated	Isomorellin	10.00^c^	18.00^c^	30.00^c^
caspase-9	Doxorubicin	10.00^c^	20.00^c^	24.00^c^
	Iso + Dox	20.00^c^	64.00^c,*,*^	73.00^c,*,*^
Procaspase-3	Isomorellin	1.00	0.93	0.84
	Doxorubicin	1.00	0.93	0.86^a^
	Iso + Dox	0.93	0.68^a,*,*^	0.67^b,*,*^
Activated	Isomorellin	8.00^c^	10.00^c^	20.00^c^
caspase-3	Doxorubicin	8.00^c^	9.00^c^	12.00^c^
	Iso + Dox	1.00	32.00^c,*,*^	36.00^c,*,*^
IκB-α	Isomorellin	1.16^a^	1.18	1.25^b^
	Doxorubicin	1.16^a^	1.17	1.21^b^
	Iso + Dox	1.17	1.21	1.40^a,*,*^
pIκB-α	Isomorellin	0.93	0.87	0.02^a^
	Doxorubicin	0.96	0.78	0.02^b^
	Iso + Dox	0.85^a^	0.73^a,*,–^	0.02^b^
NF-κB/p65	Isomorellin	1.00	0.77^a^	0.01^c^
	Doxorubicin	1.00	1.00	0.87^a^
	Iso + Dox	0.66^b^	0.02^c,*,*^	0.01^c,–,*^
MRP1	Isomorellin	0.97	0.91	0.79^b^
	Doxorubicin	0.97	0.96	0.57^b^
	Iso + Dox	0.81^a^	0.79^a,–,*^	0.35^c,*,*^

Data = The mean ratio of the density readings of target proteins normalized to β-actin from the isomorellin- and/or doxorubicin-treated to DMSO-treated cells (*n* = 3). ^a^ *P* < 0.05, ^b^ *P* < 0.01, ^c^ *P* < 0.001 *vs.* DMSO-treated cells. Comparison between isomorellin or doxorubicin treatment alone and combined drug treatment indicated by ^*,*^ *P* < 0.05 respectively, not significant difference indicated by “^–^”.

Similar results were obtained when KKU-100 cells were treated with either forbesione or doxorubicin or a combination of the two drugs. The expression of Bcl-2 decreased significantly, while that of Bax increased significantly in a dose-dependent manner, especially in the combination treatment group (Figure 4B). In cells treated with combination C3, the expression of Bcl-2 decreased 0.1-fold, which was less than the decrease observed in cells treated with doxorubicin (0.25-fold) or forbesione (0.27-fold) alone. By comparison, the expression of Bax in cells treated with combination C3 (3.24-fold) was significantly higher than that in cells treated with doxorubicin (2.17-fold) or forbesione (2.06-fold) alone (Table 4). The suppression of Bcl-2 and the enhancement of Bax resulted in a significant increase in the ratio of Bax/Bcl-2, especially in the combination groups (Table 4).

**Table 4:** **Fold-change of proteins in forbesione- and/or doxorubicin-treated** ***vs.*** **DMSO-treated KKU-100 cells**

		KKU-100 cell		
Proteins	Treatments	Fold-changes of proteins in treated ***vs.*** control cells		
		Concentration of forbesione or doxorubicin (μM)		
	Forbesione	0.25	0.50	1.00
	Doxorubicin	0.006	0.012	0.025
	Forb + Dox	C1	C2	C3
Bcl-2	Forbesione	0.63^a^	0.60^a^	0.27^c^
	Doxorubicin	0.76	0.68^b^	0.25^c^
	Forb + Dox	0.74^a^	0.65^b,–,*^	0.10^c,*,*^
Bax	Forbesione	1.56^a^	2.06^b^	2.06^c^
	Doxorubicin	1.87^a^	2.07^a^	2.17^a^
	Forb + Dox	2.35^c^	2.37^c,*,*^	3.24^c,*,*^
Bax/Bcl-2	Forbesione	0.53^b^	0.73^a^	1.63^c^
	Doxorubicin	0.84^a^	1.03	2.87^c^
	Forb + Dox	1.18	1.35^a,*,–^	12.00^c,*,*^
survivin	Forbesione	0.96	0.78^a^	0.35^c^
	Doxorubicin	1.00	0.97	0.24^c^
	Forb + Dox	0.97	0.41^a,*,*^	0.13^c,*,*^
Procaspase-9	Forbesione	0.92	0.79^a^	0.70^a^
	Doxorubicin	0.90	0.71^a^	0.65^a^
	Forb + Dox	0.69	0.68	0^c,*,*^
Activated	Forbesione	10.00^c^	39.00^c^	42.00^c^
caspase-9	Doxorubicin	22.00^c^	48.00^c^	51.00^c^
	Forb + Dox	50.00^c^	50.00^c,*,–^	64.00^c,*,*^
Procaspase-3	Forbesione	1.00	0.99	0.76^a^
	Doxorubicin	0.98	0.76	0.69^a^
	Forb + Dox	0.97	0.74^a,*,–^	0^c,*,*^
Activated	Forbesione	9.00^c^	9.00^c^	42.00^c^
caspase-3	Doxorubicin	20.00^c^	40.00^c^	50.00^c^
	Forb + Dox	15.00^c^	47.00^c,*,–^	54.00^c,*,*^
IκB-α	Forbesione	1.05	1.07	1.15
	Doxorubicin	1.03	1.06	1.12
	Forb + Dox	1.16^a^	1.21^b,–,*^	1.21^b,*,*^
pIκB-α	Forbesione	0.97	0.97	0.14^b^
	Doxorubicin	0.58^a^	0.58^a^	0.14^b^
	Forb + Dox	0.25^c^	0.01^c,*,*^	0.01^c,*,*^
NF-κB/p65	Forbesione	1.00	0.78^b^	0.12^b^
	Doxorubicin	1.00	1.00	1.00
	Forb + Dox	0.95	0.01^c,*,*^	0.01^c,*,*^
MRP1	Forbesione	0.89^a^	0.79^a^	0.71^a^
	Doxorubicin	1.00	0.96	0.82^a^
	Forb + Dox	0.75^a^	0.74^b,–,*^	0.34^c,*,*^

Data = The mean ratio of the density readings of target proteins normalized to β-actin from the forbesione- and/or doxorubicin-treated to DMSO-treated cells (*n* = 3). ^a^ *P* < 0.05, ^b^ *P* < 0.01, ^c^ *P* < 0.001 *vs.* DMSO-treated cells. Comparison between forbesione or doxorubicin treatment alone and combined drug treatment indicated by ^*,*^ *P* < 0.05 respectively, not significant difference indicated by “^–^”.

Isomorellin, forbesione or doxorubicin alone suppressed the expression levels of survivin and procaspase-9 and procaspase-3, while those treatments augmented the expression of activated caspase-9 and caspase-3 in a dose-dependent manner. The fold change of the combined treatment (C3), compared to individual treatments, was significantly augmented (Figures 4A & B and Tables 3 &4).

### Isomorellin/doxorubicin and forbesione/doxorubicin treatments suppress NF-кB/p65 activation in CCA cell lines

**Figure 5:**
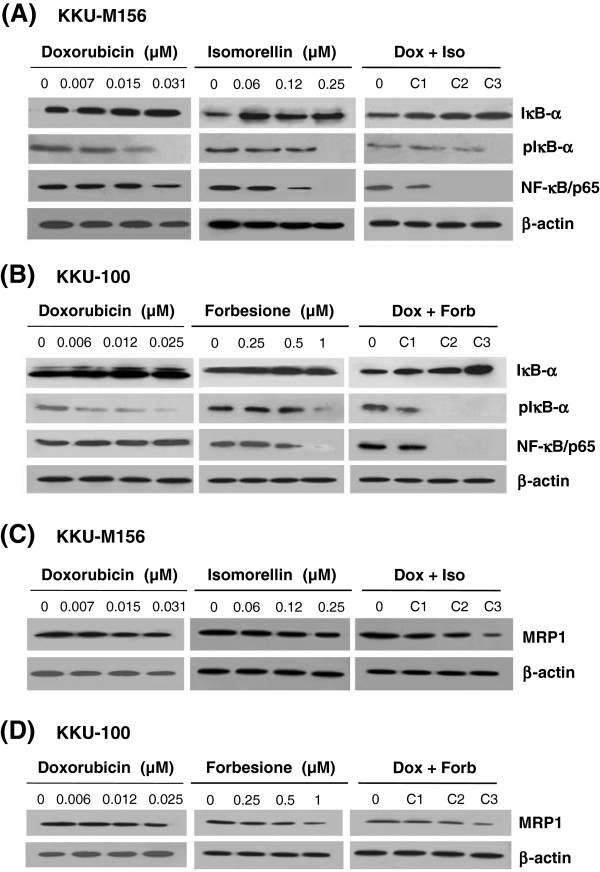
**Effect of isomorellin, forbesione and/or doxorubicin on protein expression in cholangiocarcinoma cell lines. A** and **C**, KKU-M156 cells treated with DMSO or indicated concentrations of isomorellin and/or doxorubicin; **B** and **D**, KKU-100 cells treated with DMSO or indicated concentrations of forbesione and/or doxorubicin for 48 h. Protein expression of nuclear factor-κB (NF-κB), inhibitor of NF-κB (IκB-α), phosphorylated IκB-α (pIκB-α) and multidrug resistance-associated protein 1 (MRP1) was determined by Western blot analysis.

NF-кB is known to activate the expression of genes involved in cell survival (anti-apoptotic proteins: Bcl-2, survivin) [33] and its down-regulation is associated with an apoptotic response [34]. The effects of isomorellin, forbesione, doxorubicin and their combinations on the expression of NF-кB/p65 in KKU-M156 cells were examined using Western blot analysis. Isomorellin or doxorubicin alone significantly suppressed NF-кB/p65 protein expression, and the combined treatment (C2) was even more effective (0.02-fold) than the individual treatments (Figure 5A and Table 3). We further examined whether an inhibitor of NF-кB (IкB-α) and phosphorylated IкB-α (pIкB-α) might be involved in the inhibition of NF-кB activation. IкB-α expression was slightly increased following isomorellin (1.25-fold) or doxorubicin (1.21-fold) treatment, and the combined treatment (C3) (1.40-fold) was found to be more effective (Figure 5A and Table 3). In contrast, the pIкB-α protein level was significantly decreased in cells treated with isomorellin or doxorubicin alone or their combination (Figure 5A and Table 3).

Similar results were obtained when KKU-100 cells were treated with either forbesione or doxorubicin alone or in combination. Forbesione, doxorubicin or their combination significantly enhanced IкB-α protein expression while suppressing pIкB-α and NF-кB/p65 (Figure 5B). The increase in IкB-α expression (1.21-fold) and the decrease in pIкB-α (0.01-fold) and NF-кB/p65 expression (0.01-fold) were significantly higher in cells treated with combination C3 than in those treated with the single drug (Figure 5B and Table 4).

### Isomorellin/doxorubicin and forbesione/doxorubicin treatments down-regulate expression of the drug-efflux transporter protein (MRP1) in CCA cell lines

The p-glycoprotein and multidrug resistance proteins (MRP) 1–3 are membrane pumps expressed on cholangiocytes [14, 15], and their overexpression is associated with drug resistance. To determine whether isomorellin, forbesione, doxorubicin or their combinations modulate the cellular efflux pathway, we evaluated the expression of MRP1, the specific membrane pump. In KKU-M156 cells, doxorubicin, isomorellin, or their combination (C3) significantly suppressed the expression of the MRP1 protein by 0.57-, 0.79-, and 0.35-fold compared with the control, respectively (Figure 5C and Table 3). The suppressive effect on MRP1 expression in the combination treatment (C3) was significantly greater than in the single drug treatments (Table 3).

Similarly, MRP1 protein expression in KKU-100 cells was significantly decreased by treatments with doxorubicin or forbesione alone or their combination (C3). This effect was significantly greater in the combination (C3) treatment than in the single drug treatments (Figure 5D and Table 4).

## Discussion

Chemotherapy has been used for the treatment of advanced CCA, but patient outcomes are not successful in most cases due to drug resistance and severe side effects. Accordingly, the combination of chemotherapeutic agents and natural compounds with distinct molecular mechanisms is considered a promising therapeutic strategy with higher clinical efficacy and better patient survival than traditional therapy [35]. Many caged xanthones isolated from *G. hanburyi* Hook.f. (family Guttiferae) have inhibitory activity against the growth of various human cancer cell lines [29, 36] as well as anti-cancer and anti-tumor activities [37]. We recently demonstrated that four caged xanthones, isomorellin, isomorellinol, forbesione and gambogic acid, can induce cell cycle arrest [30] and apoptotic cell death in CCA cell lines [29].

In the present study, we investigated the combined effects of two caged xanthones (isomorellin and forbesione) with doxorubicin in the CCA KKU-100, KKU-M139 and KKU-M156 cell lines using the median-effect equation and the CI method. Because the CCA cell lines used in this study were derived from intrahepatic CCA, which comes in close contact with normal hepatocytes, Chang (normal hepatocyte) cells were used as controls for the selective cytotoxicity study. We found that isomorellin, forbesione and doxorubicin alone mediated significant inhibitory effects on the growth of Chang cells and KKU-100, KKU-M139 and KKU-M156 cells in a dose-dependent manner. The inhibitory effects of isomorellin, forbesione and doxorubicin on cell growth were greater in KKU-M156 cells than in KKU-M139 and KKU-100 cells, possibly due to the different histologic types and drug sensitivities of these three cell lines [14]. The two caged xanthones (isomorellin and forbesione) showed selective growth inhibitory activity in cancer cells compared with Chang cells. These results confirm our previous study, which showed that isomorellin and forbesione selectively inhibit the growth of CCA cell lines compared to normal human peripheral blood mononuclear cells [29]. The IC_50_ values of isomorellin and forbesione reported for KKU-100 and KKU-M156 cells in this study differed from those reported previously [29]. This may be due in part to the different passage numbers of the cell lines. In the previous study, cells were used at the 20^th^-27^th^ passage, while in the present study cells were used at the 70^th^-76^th^ passage. Similar to our previous results, the susceptibility of four human melanoma cell lines to anthracyclines was much higher at early passages than at later passages [38]. These results suggest that the activity of some molecules involved in chemosusceptibility might be gradually lost during serial passaging.

**Figure 6:**
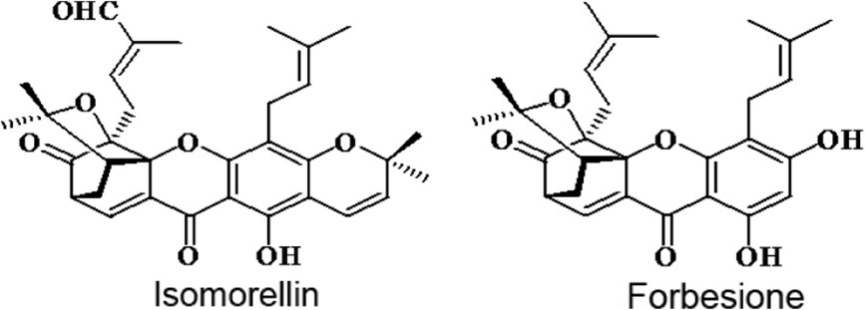
**Chemical structures of the two caged xanthones.**

Doxorubicin is a well-known cancer therapeutic agent but is highly toxic in normal tissues during cancer therapy [39]. Doxorubicin has harmful effects on health and can induce primary and secondary drug resistance in tumor cells, limiting the success of cancer chemotherapy [5, 6]. Combination chemotherapy is a superior modality of therapy, especially when naturally occurring dietary supplements (with known anticancer activity) are used to reduce the systemic toxicity of chemotherapy [40, 41]. In this study, the isomorellin/doxorubicin combination synergistically enhanced the growth inhibition of KKU-M139 and KKU-M156 cells. A synergistic effect was also demonstrated after treatment with the forbesione/doxorubicin combination in KKU-100 and KKU-M139 cells. These combinations also showed an antagonistic effect in Chang cells. From the calculated CI values, the combination of isomorellin/doxorubicin showed the highest synergistic effect in KKU-M156 cells (CI value at IC_90_ = 0.24), whereas the combination of forbesione/doxorubicin showed the highest synergistic effect in KKU-100 cells (CI value at IC_90_ = 0.39). According to these results, the effects of isomorellin/doxorubicin in KKU-M156 cells and of forbesione/doxorubicin in KKU-100 cells were selected for further investigation. These results show that the interaction between each caged xanthone and doxorubicin differed in each CCA cell line. This difference may be due to the different chemical structures of the two compounds; although they share a caged structure, a chromene ring is present in isomorellin but absent in forbesione. Forbesione has two non-functional prenyl side-chains, whereas one of the two prenyl side-chains of isomorellin is functionalized as an aldehyde (Figure 6). Our results provide corroborative evidence for the functional differences between isomorellin and forbesione in terms of their inhibition of growth in three CCA cell lines. In addition, the calculated DRIs demonstrated that the combination of isomorellin/doxorubicin can reduce the effective dose of doxorubicin for KKU-M156 cells at IC_75_ and IC_90_ by 2.18-fold and 4.23-fold, respectively, and for KKU-M139 cells at IC_75_ and IC_90_ by 2.57-fold and 2.17-fold, respectively (Table 2). Similarly, the combination of forbesione/doxorubicin could reduce the effective dose of doxorubicin for KKU-100 cells at IC_75_ and IC_90_ by 1.73-fold and 2.99-fold, respectively, and for KKU-M139 cells at IC_75_ and IC_90_ by 5.20-fold and 4.12-fold, respectively (Table 2). These results indicate the broad spectrum of the anti-CCA effects of these two combinations. These findings support the hypothesis that combinations of plant compounds and chemotherapeutic drugs can reduce the concentration of doxorubicin used in treatment, retaining its benefits and minimizing its cytotoxic effects while enhancing therapeutic efficacy.

Although 5-FU has been used widely for chemotherapy in patients with CCA [4], our preliminary data show that the combination of caged xanthones with 5-FU had an antagonistic effect on the inhibition of the growth of CCA cell lines (data not shown).

Chemotherapeutic drugs indirectly induce apoptosis through DNA damage and cell cycle arrest. Doxorubicin induces tumor cell apoptosis by intercalating into DNA and inhibiting topoisomerase II [42]. Resistance to anticancer agents is thought to be the failure of these agents to induce apoptosis [43, 44]. Therefore, agents that can induce apoptosis should improve therapeutic efficacy. Isomorellin and forbesione are reported to induce cell cycle arrest at G0/G1 phase [30] and to induce apoptosis in KKU-100 and KKU-M156 cells [29]. The synergistic effect of gambogic acid with 5-FU in the induction of apoptosis was reported in human gastric carcinoma BGC-823 cells [17]. In this respect, we found that isomorellin and forbesione enhanced apoptosis induced by doxorubicin in KKU-M156 and KKU-100 cells, respectively (Figure 3).

Several chemotherapeutic drugs induce apoptosis through a mitochondrial pathway regulated by the Bcl-2-family of proteins, including pro-apoptotic proteins (i.e., Bax) and anti-apoptotic proteins (i.e., Bcl-2) [44]. In the current study, treating KKU-M156 cells with isomorellin and/or doxorubicin or treating KKU-100 cells with forbesione and/or doxorubicin increased the expression of Bax while decreasing the expression of Bcl-2, leading to an increase in the Bax/Bcl-2 ratio (Figure 4 and Tables 3 &4). Our results confirm those of previous studies in which isomorellin, forbesione and gambogic acid induced apoptosis through the regulation of Bax (up) and Bcl-2 (down) protein expression in the human gastric carcinoma cell line MGC-803 [19] and in CCA cell lines [29].

In the intrinsic apoptotic pathway, an increase in the Bax/Bcl-2 ratio leads to the release of cytochrome C from mitochondria; the released cytochrome C then forms a complex with apoptotic protease activating factor-1 (Apaf-1) and procaspase-9 (called the apoptosome), leading to the activation of caspase-9 and caspase-3 and subsequent apoptotic cell death [45, 46]. Survivin is a human inhibitor of apoptosis proteins (IAP) and inhibits activated caspase-3 and caspase-7 [47]. In the current study, treatment of KKU-M156 cells with isomorellin and/or doxorubicin and treatment of KKU-100 cells with forbesione and/or doxorubicin decreased the protein expression of survivin, procaspase-9 and procaspase-3, while those treatments increased protein expression levels of activated caspase-9 and caspase-3. These effects were more prominent following combination treatment (Figure 4 and Tables 3 &4), suggesting that these treatments induced apoptosis in both CCA cell lines through caspase-9 and caspase-3 activation. Isomorellin and forbesione induced apoptosis by decreasing survivin protein expression while increasing the activation of caspase-9 and caspase-3 in CCA cells [29]. These results suggest that the synergistic induction of apoptosis observed following combination treatment is mediated through a caspase-dependent mitochondrial apoptosis pathway.

Alongside the induction of apoptosis, anti-cancer drugs can suppress tumor growth via the suppression of NF-κB activation. NF-κB activates the expression of cell survival regulation genes (anti-apoptotic proteins Bcl-2 and survivin) and drug resistance genes [33]. NF-κB comprises p50 and p65 subunits that form an inactive complex with the inhibitor protein IκB in the cytoplasm. Following cellular stimulation, IκB and NF-κB/p65 are phosphorylated by IκB-α kinase (IKK), leading to IκB degradation [33] and the DNA binding of NF-κB/p65, which initiates the transcription of its target genes [33, 48]. Doxorubicin inhibits NF-κB activation in various cell lines [49]. In our previous study, we found that isomorellin inhibits NF-κB activation in KKU-100 and KKU-M156 cells by increasing the level of IκB-α in the cytosol and decreasing the expression and nuclear translocation of NF-κB/p65 protein [30]. Therefore, we theorized that combined treatment involving caged xanthones and drugs could suppress the NF-κB pathway. Our data show that the combination of isomorellin or forbesione with doxorubicin strongly suppressed NF-κB activation by decreasing the expression of NF-κB/p65, reducing the level of pIκB-α, and increasing the level of IκB-α (Figures 5A & B and Tables 3 &4). The inhibition of NF-κB activation by combination treatment was confirmed by a decrease in the protein expression levels of Bcl-2 and survivin, which are regulated by NF-κB at the transcriptional level [33].

The enhanced expression of MDRs and MDR-associated proteins (MRPs) in cancer cells is a major cause of multidrug resistance both *in vitro* and *in vivo*[13]. P-glycoprotein (P-gp) and MRP1-3 are known to be overexpressed in CCA [14, 15]. A number of natural products are known to enhance the drug-induced cytotoxicity of cancer cells by modulating drug efflux pathways, such as by decreasing the expression of the P-gp, MRP1 and MRP2 membrane efflux pumps [50, 51]. Our results demonstrated that the combination of isomorellin or forbesione with doxorubicin strongly suppressed MRP1 protein expression compared to treatment with either of the compounds or with drug treatment alone (Figures 5C & D and Tables 3 &4). The observed decrease in MRP1 protein expression may therefore enhance drug-induced cytotoxicity by increasing intracellular drug concentrations.

In this study, we used CCA cells derived from primary tumors of OV-associated CCAs. It is known that the gene expression profiles of OV-associated intrahepatic cholangiocarcinomas (ICCs) in Thai patients are different from those of non-OV-associated ICCs in Japanese patients [52]. Genes involved in xenobiotic metabolism are overexpressed in OV-associated ICCs, whereas genes related to growth factor signaling pathways are overexpressed in non-OV-associated ICCs. Using exome sequencing, distinct mutational patterns between OV-related CCAs from Thailand and non-OV related CCAs from Singapore (Asia) and Romania (Europe) were reported [53]. *BAP1, IDH1* and *IDH2* were more frequently mutated in non-OV related CCAs, whereas *TP53* mutations showed a reciprocal pattern. As these differences might affect the sensitivities of CCAs to chemotherapeutic agents, further study is required to assess the applicability of the combination of isomorellin or forbesione with doxorubicin for the treatment of non-OV related CCAs.

## Conclusions

Our results show that isomorellin and forbesione in combination with doxorubicin can synergistically inhibit growth and induce apoptosis in KKU-100, KKU-M139 and KKU-M156 cells. The mechanisms most likely affected by this treatment are the modulation of apoptosis-regulated protein expression, the inactivation of NF-κB and the down-regulation of MRP1 protein expression. Using the same combinations of drugs, toxicity was not induced in normal liver cells (Chang cells), suggesting the feasibility of using these caged xanthones as an adjunct to chemotherapy for the management of OV-associated CCAs. We also provide a rationale for further *in vivo* and clinical studies to determine the effectiveness of these combinations in the treatment of CCA.

## Methods

### Plant compounds and chemotherapeutic drug

Two caged xanthones, isomorellin and forbesione (Figure 6), were isolated from *G. hanburyi* Hook.f. (family Guttiferae) [36]. An 8 mM working solution in DMSO was prepared before use. Injectable doxorubicin (RegNo. 1C 47/50, Batch No. 7AA014, 2 mg/ml) was kindly provided by Fresenius Kabi Oncology Ltd. (Hayarna, India). These compounds and the drug were diluted to the required final concentrations prior to their use.

### Cell culture

CCA KKU-100, KKU-M139 and KKU-M156 cells were isolated from Thai CCA patients [14, 54]. Normal human liver (Chang) cells were purchased from CLS Cell Lines Service GmbH (Eppelheim, Germany). Cells were maintained at 37°C in a humidified atmosphere containing 5% CO_2_ in RPMI-1640 medium (Gibco BRL, Grand Island, NY, USA) supplemented with 10% fetal bovine serum, 100 units/ml penicillin and 100 μg/ml streptomycin (Gibco BRL).

### *In vitro*cytotoxicity assay

Cells (1 x 10^5^ cells/well) were seeded in 96-well plates for 24 h. Cells were treated for 72 h with compounds or drug by adding 100 μl/well of each concentration in triplicate to obtain a final concentration of 0.25-128 μM/well for each caged xanthone and 0.004-32 μM/well for doxorubicin. Cells treated with 0.1% DMSO were used as a control. Cell growth was determined using the sulforhodamine B (SRB) assay [55]. The 50% growth inhibitory concentrations (IC_50_) of the compounds or drug used on KKU-100, KKU-M139, KKU-M156 and Chang cells were calculated from concentration-effect curves after linear regression analysis. The selectivity of each compound was determined by its toxicity to each cancer cell line compared to the Chang cells (i.e., vis-à-vis the respective IC_50s_).

### Evaluation of drug interaction

For combination studies, KKU-100 cells were treated with isomorellin (0.1, 0.2, 0.4, 0.8 and 1.6 μM) and/or doxorubicin (0.0078, 0.0156, 0.0313, 0.0625 and 0.125 μM) or with forbesione (0.0313, 0.0625, 0.125, 0.25, 0.5, 1 and 2 μM) and/or doxorubicin (0.0078, 0.0156, 0.0313, 0.0625, 0.125, 0.25 and 0.5 μM). KKU-M139 cells were treated with isomorellin (0.125, 0.25, 0.5, 1, 2 and 4 μM) and/or doxorubicin (0.0625, 0.125, 0.25, 0.5, 1 and 2 μM) or with forbesione (0.125, 0.25, 0.5, 1 and 2 μM) and/or doxorubicin (0.0625, 0.125, 0.25, 0.5 and 1 μM). KKU-M156 cells were treated with isomorellin (0.0313, 0.0625, 0.125 and 0.25 μM) and/or doxorubicin (0.0391, 0.0781, 0.1563 and 0.3125 μM) or with forbesione (0.0625, 0.125, 0.25, 0.5 and 1 μM) and/or doxorubicin (0.0391, 0.0781, 0.1563, 0.3125 and 0.625 μM). After 72 h, cell growth was examined using the SRB assay [55]. The Chou *et al*. median-effect analysis [31] and the Chou and Talalay combination index (CI) method [32] were used to determine the synergy, additivity, or antagonism of the compound-drug combinations [55]. A combination index (CI) of < 1, = 1 or > 1 indicates synergy, additivity or antagonism, respectively. The dose-reduction index (DRI) was defined as the degree of dose reduction possible in a combination for a given degree of effect compared with the dose of each drug alone [55].

### Ethidium bromide/Acridine orange (EB/AO) staining

Apoptosis was determined by the detection of nuclear morphology using ethidium bromide/acridine orange (EB/AO) staining [56]. KKU-100 and KKU-M156 cells (1 x 10^4^ cells/well) were grown in 96-well plates at 37°C for 24 h and then treated with the studied compounds combined with doxorubicin at concentrations that showed a synergistic inhibitory effect on growth for 48 h. Cells treated with the same concentrations of the compounds or with doxorubicin alone were also examined. Cells cultured with 0.1% DMSO were used as controls. The treated cells were stained with an EB/AO mixture (Sigma Chemical, St. Louis, MO, USA) and observed under a Nikon fluorescent microscope. The percentage of apoptotic cells with condensed chromatin or fragmented chromatin was calculated from 500 counted cells.

### Protein extraction and Western blot analysis

Cells (1 x 10^6^) were seeded into 10-cm dishes for 24 h and then treated with 0.1% DMSO or the appropriate concentrations of the studied compounds and doxorubicin, either alone or in combination, for 48 h. Protein extraction and Western blot analysis were performed [30]. After blocking, the membranes were incubated with primary antibodies against Bcl-2, Bax, survivin, procaspase-9, procaspase-3, IκB-α, pIκB-α, NF-κB/p65, MRP1 (Santa Cruz Biotechnology, Santa Cruz, CA, USA), activated caspase-9 (Cell Signaling, Beverly, MA, USA), activated caspase-3 or β-actin (Sigma Chemical) at 4°C overnight. The membranes were incubated at room temperature for 1 h with the horseradish peroxidase-conjugated goat anti-mouse IgG and goat anti-rabbit IgG antibodies (Santa Cruz Biotechnology). The blots were detected by enhanced chemiluminescence (Pierce Biotechnology, Rockford, IL, USA) and fluorogrammed with CL-XPosure film. The intensities of the protein bands were quantified using Scion Image Software. The relative intensities were evaluated and normalized to β-actin.

### Statistical analyses

Data are expressed as the mean ± standard error (SE). Student’s *t*-test was used to compare untreated control cells with cells treated with compounds and drug alone or in combination. Differences were considered significant at ^a^*P* < 0.05, ^b^*P* < 0.01 and ^c^*P* < 0.001. All analyses were performed using Microsoft Excel (Microsoft, Redmond, WA, USA).

## Abbreviations

CCA: Cholangiocarcinoma
SRB: Sulforhodamine B
CI: Combination index
MDR: Multidrug resistance protein families
MRPs: MDR-associated proteins
P-gp: P-glycoprotein
5-FU: 5-fluorouracil
IC_50_: 50% growth inhibitory concentrations
IC_75_: 75% growth inhibitory concentrations
IC_90_: 90% growth inhibitory concentrations
DRI: Dose-reduction index
EB/AO: Ethidium bromide/acridine orange
NF-κB: Nuclear factor-kappa B
IκB-α: Inhibitor of NF-кB
pIκB-α: Phosphorylated IкB-α
Apaf-1: Apoptotic protease activating factor-1.
